# A resected case of liver metastases from extra-adrenal retroperitoneal paraganglioma with von Recklinghausen’s disease 16 years after the initial surgery

**DOI:** 10.1186/s40792-015-0089-2

**Published:** 2015-09-21

**Authors:** Kenji Sakai, Yoshito Tomimaru, Hidetoshi Eguchi, Shigeru Marubashi, Akira Tomokuni, Tadafumi Asaoka, Hiroshi Wada, Koichi Kawamoto, Koji Umeshita, Yuichiro Doki, Masaki Mori, Hiroaki Nagano

**Affiliations:** Department of Surgery, Graduate School of Medicine, Osaka University, 2-2 Yamadaoka E-2, Suita, 565-0871 Osaka Japan; Division of Health Sciences, Graduate School of Medicine, Osaka University, Suita, Osaka Japan

**Keywords:** von Recklinghausen’s disease, Paraganglioma, Liver metastasis, Hepatectomy

## Abstract

The patient was a 46-year-old man who had undergone resection for a bulky retroperitoneal tumor 16 years previously during a follow-up for von Recklinghausen’s disease. Histopathological examination of the resected specimen showed that the tumor was an extra-adrenal paraganglioma. After the surgery, he had survived without any recurrence of the tumor. However, 16 years after the initial surgery, liver tumors were identified, and he was referred to our hospital for further investigation and treatment. Abdominal imaging modalities showed three masses in the left lateral segment of the liver. Fluorodeoxyglucose-positron emission tomography/computed tomography showed an abnormal uptake of fluorodeoxyglucose corresponding to the mass lesions. The patient was diagnosed with a metastatic paraganglioma based on histopathological examination of a liver mass biopsy. The patient underwent left lateral sectionectomy of the liver. Histopathological examination of the resected specimen revealed proliferating cells with basophilic cytoplasm and oval densely stained nuclei arranged in an alveolar pattern, which was similar to the findings of the initial resection specimen. Immunohistochemical staining was positive for synaptophysin and chromogranin A. Based on these findings, the resected tumors were histopathologically diagnosed with liver metastases from the retroperitoneal paraganglioma. We concluded that this is an extremely rare case of liver metastases occurring long after the initial resection of extra-adrenal peritoneal paraganglioma with von Recklinghausen’s disease.

## Background

Although the association of pheochromocytoma with von Recklinghausen’s disease is well recognized, the association of paraganglioma (extra-adrenal pheochromocytoma) with von Recklinghausen’s disease is rare [1, 2]. To the best of our knowledge, there are no reported cases of resection of liver metastases after the surgery for primary paraganglioma in patients with von Recklinghausen’s disease. We encountered a case of liver metastases from a retroperitoneal paraganglioma in a patient with von Recklinghausen’s disease who had undergone a hepatectomy, 16 years after the initial surgery. We herein report this case and review the literature.

## Case presentation

### Patient

The patient was a 46-year-old man.

### Chief complaint

None.

### Past history

At the age of 45, the patient was diagnosed with hypertension and cerebral aneurysm and was being followed up without treatment.

### History of present illness

**Fig. 1:**
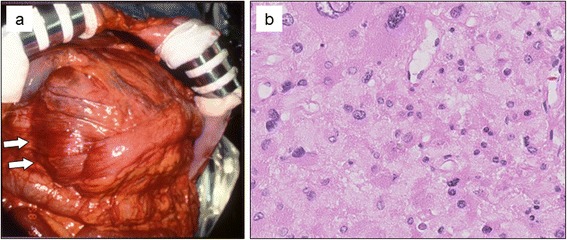
Findings at the initial laparotomy. A bulky mass with hemorrhagic necrosis, measuring about 25 × 17 × 15 cm is observed in the retroperitoneum (**a**; *arrow*). Proliferating cells with weak basophilic cytoplasm and oval densely stained nuclei arranged in an alveolar pattern were histopathologically observed in the tumor, and the tumor was histopathologically diagnosed as extra-adrenal retroperitoneal paraganglioma (**b**)

Sixteen years ago (at the age of 30), the patient was found to have a bulky retroperitoneal paraganglioma measuring 20 cm during a follow-up of von Recklinghausen’s disease and underwent surgery in our department. The tumor, which had hemorrhagic necrosis, measured 25 × 17 × 15 cm and was located in the retroperitoneum (Fig. 1a). The tumor was resected together with the inferior vena cava and the common hepatic artery. Proliferating cells with weak basophilic cytoplasm and oval densely stained nuclei arranged in an alveolar pattern were histopathologically observed in the tumor, resulting in the diagnosis of extra-adrenal retroperitoneal paraganglioma (Fig. 1b). After the surgery, the patient was followed up on an outpatient basis. Six years after the surgery, he moved to another area and stopped visiting the hospital using his own judgment. At the age of 45, the patient underwent examination for hypertension and cerebral aneurysm and was found to have liver masses. Therefore, he was referred to our hospital for further investigation and treatment.

### Physical examination

His blood pressure was 115/64 mmHg under antihypertensive medication, and he did not show any remarkable findings. A surgical scar was observed in the upper abdomen. There were café au lait spots all over the body and neurofibromatosis of the skin.

### Blood test results

The laboratory findings, including the total catecholamine level, urinary vanillylmandelic acid level, urinary homovanillic acid level, and levels of tumor markers (carcinoembryonic antigen, carbohydrate antigen 19-9, alpha-fetoprotein, des-gamma-carboxyl prothrombin, and neuron-specific enolase) were within normal limits.

### Abdominal ultrasonography

Three space-occupying lesions with high echo level (measuring 2.5, 1.5, and 1.5 cm in size) were observed in the left lateral segment of the liver.

### Abdominal computed tomography

**Fig. 2:**
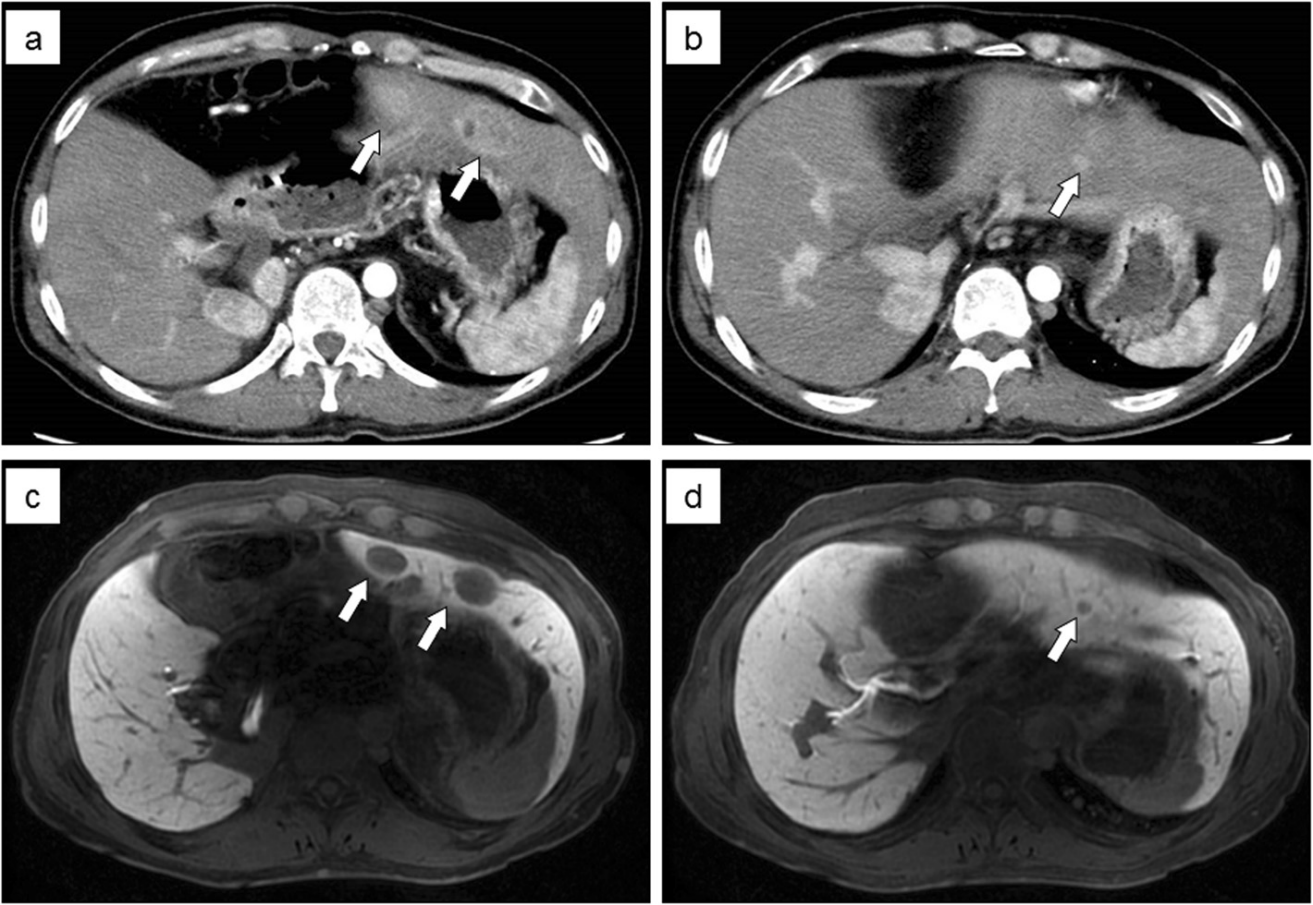
Enhanced abdominal computed tomography (CT) and magnetic resonance imaging (MRI). CT images demonstrates that three hypervascular masses, the largest of which is 2.9 cm in size, are located in the left lateral segment of the liver and show early staining and washout in the portal-venous phase (**a**, **b**; *arrow*). Three masses in the left lateral segment of the liver show no uptake of the contrast agent in the hepatocyte phase of MRI (**c**, **d**; *arrow*)

Plain and enhanced computed tomography (CT) showed that three hypervascular masses, the largest of which was 2.9 cm in size, were located in the left lateral segment of the liver. Plain CT showed low-density areas. Contrast-enhanced CT showed early staining and washout in the portal-venous phase (Fig. 2a, b).

### Abdominal magnetic resonance imaging

Gadolinium-ethoxybenzyl diethylenetriamine pentaacetic acid-enhanced magnetic resonance imaging (MRI) showed that three masses with marginal enhancement were located in the left lateral segment of the liver, with no uptake of the contrast agent in the hepatocyte phase (Fig. 2c, d).

### Fluorodeoxyglucose-positron emission tomography/CT

Abnormal uptake of fluorodeoxyglucose (FDG; maximum standardized uptake value 2.7) was observed in the lesions identified by the above imaging studies. There was no significant abnormal FDG uptake in other regions.

### Biopsy of liver mass

Liver mass biopsy was performed for a definite diagnosis. Proliferating cells with weak basophilic cytoplasm and oval densely stained nuclei arranged in an alveolar pattern, similar to the findings of the initial resection paraganglioma specimen, were observed. Immunohistochemical staining was positive for synaptophysin and chromogranin A and negative for HAS and cytokeratin AE1/AE3. Based on these findings, the patient was diagnosed with liver metastases of the extra-adrenal retroperitoneal paraganglioma.

### Operative findings

**Fig. 3:**
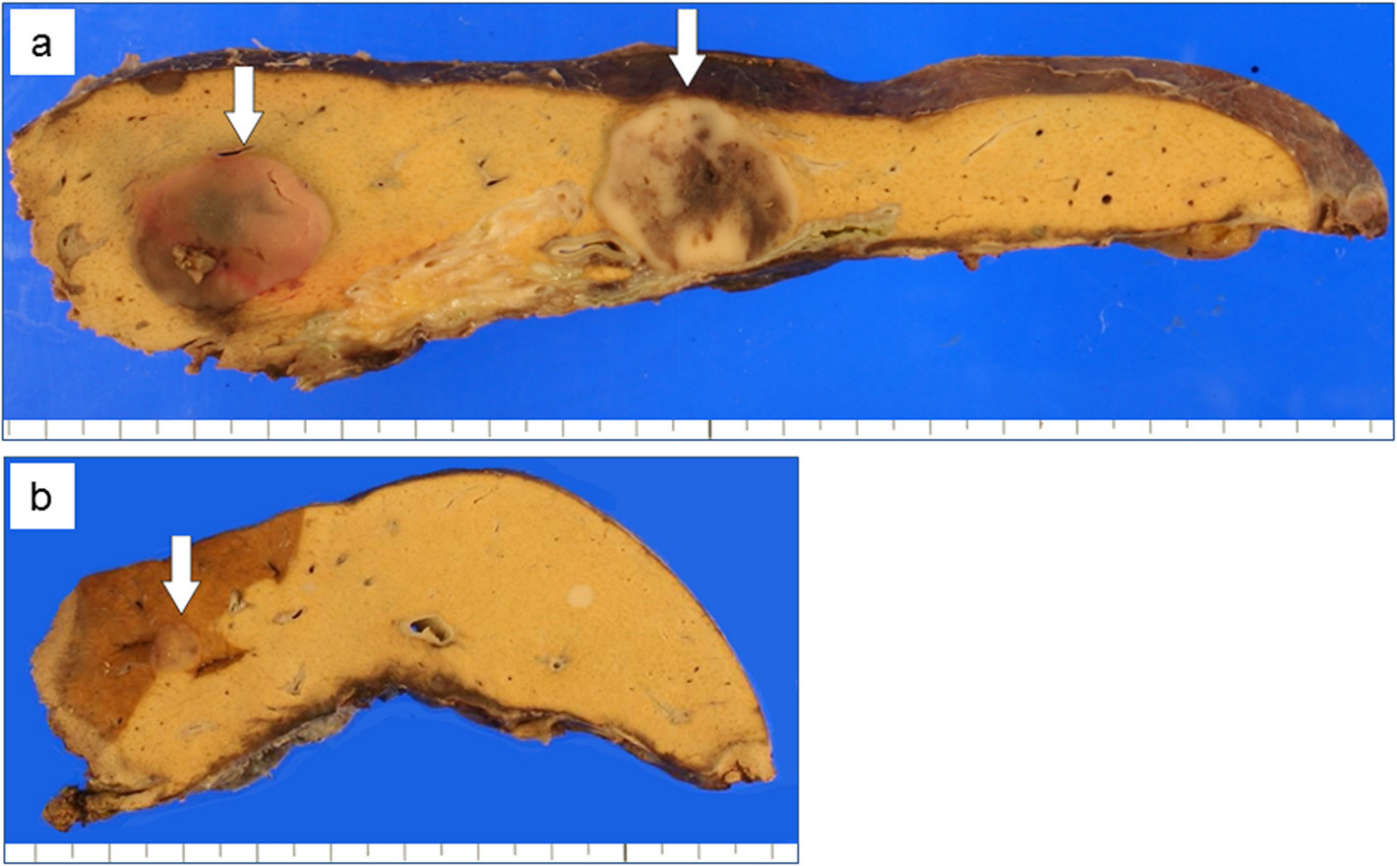
Macroscopic findings of resected specimen. Whitish well-circumscribed solid tumors with capsules and no invasion of the surrounding tissues are observed (**a**, **b**; *arrow*)

The patient underwent left lateral sectionectomy for the liver metastases from the paraganglioma. A laparotomy using an upper abdominal median incision plus high transverse incision was performed under general anesthesia. The adhesion from the previous surgery was separated. Three tumors were confirmed by palpation and intraoperative ultrasonography. There were no other metastatic lesions. The left lateral segment of the liver was resected. Macroscopic examination of the resected specimen showed whitish well-circumscribed solid tumors with capsules, with no invasion of the surrounding tissues (Fig. 3).

### Histopathological findings

**Fig. 4:**
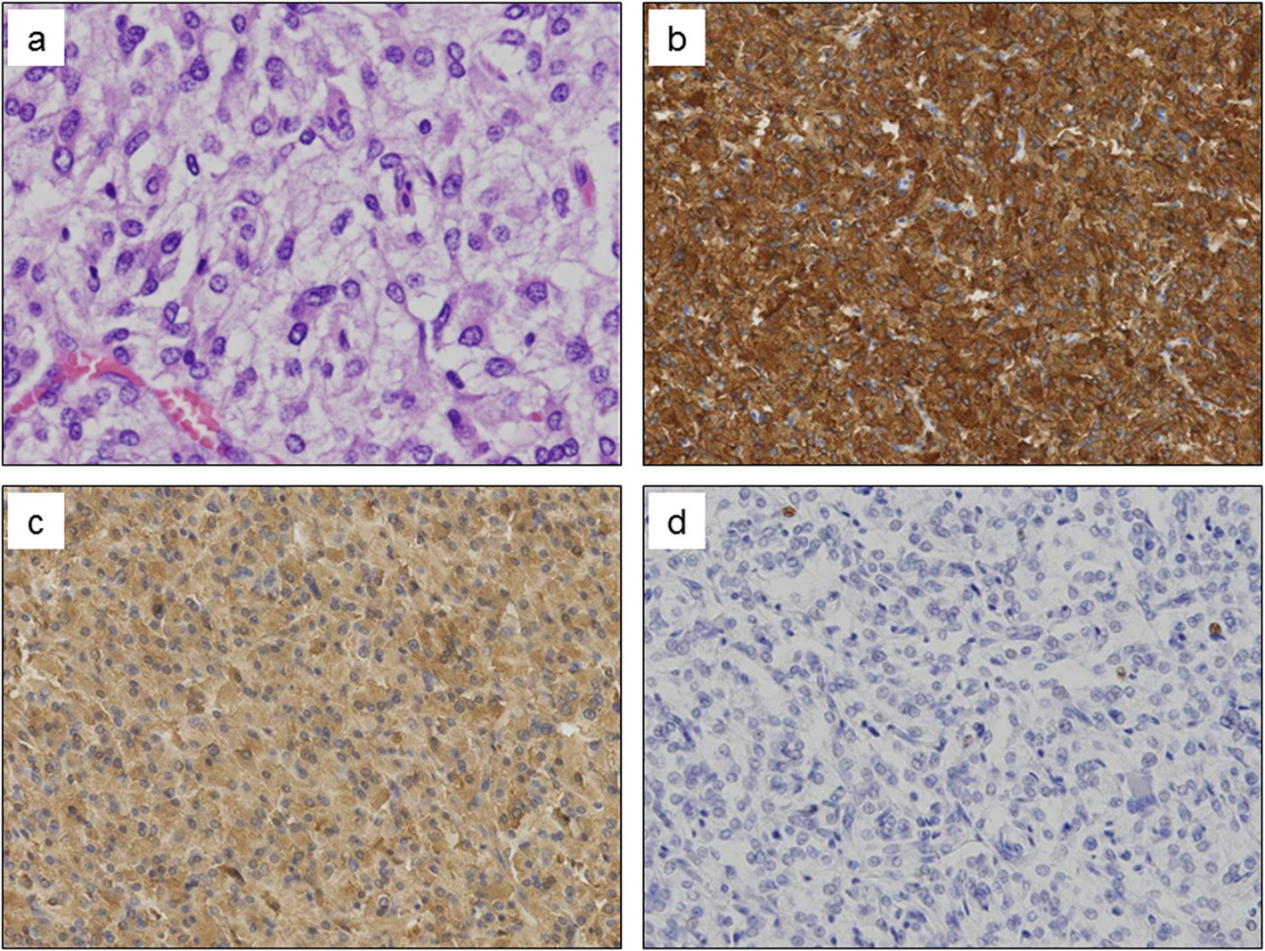
Histopathological findings (×200). Hematoxylin and eosin staining shows that although the Zellballen pattern typical of paraganglioma is not apparent, the tumors are composed of large and irregular nests of cells with anisokaryosis and basophilic cytoplasm, resulting in the final diagnosis of liver metastases from the paraganglioma (**a**). Immunohistochemical staining is positive for synaptophysin (**b**) and chromogranin A (**c**). The MIB-1 index is less than 0.5 % (**d**)

Although the Zellballen pattern typical of paraganglioma was not apparent, the tumors were composed of large and irregular nests of cells with anisokaryosis and basophilic cytoplasm (Fig. 4a), which was similar to the findings from the initial retroperitoneal paraganglioma resection specimen. Immunohistochemical staining was positive for synaptophysin (Fig. 4b) and chromogranin A (Fig. 4c), and negative for CAM 5.2. The MIB-1 (antibody against Ki-67) index was below 0.5 % (Fig. 4d). The final diagnosis was liver metastases from a well-differentiated paraganglioma.

### Postoperative outcome

After the surgery, the patient had a favorable clinical course without complications. At the time of this report (6 months after the surgery), the patient has experienced no relapses and is being followed up on an outpatient basis.

## Discussion

von Recklinghausen’s disease is an autosomal dominant disease first described by von Recklinghausen’s in 1882 [3]. The major clinical features include multiple neurofibromas and café au lait spots in the skin. The other common clinical features include deformity of the spinal column and rib cage, eye disease, and neural tumor of the central nervous system. The incidence is about one in 3000 to 4000 live births. Although von Recklinghausen’s disease is a genetic disorder, only about 30 % of patients have a family history of the condition. Mutations in the rest of the patients (about 70 %) appear to be spontaneous mutations [4]. Broadly, tumors arising from chromaffin tissue in the sympathetic ganglia are referred to as pheochromocytomas, although strictly speaking, only those of adrenal origin are pheochromocytomas, whereas those of extra-adrenal origin are paragangliomas. Paragangliomas occur more commonly in the retroperitoneum, accounting for 1.8 to 2.1 % of all retroperitoneal tumors [5–7]. A study reported that 14 to 50 % of retroperitoneal paragangliomas are malignant [8]. Evidence suggests that the prevalence of pheochromocytoma (those of adrenal origin) in patients with von Recklinghausen’s disease is relatively high [9, 10]. According to a study by Walther et al., the incidence was 0.1 to 5.7 % [11], considered to be approximately 10 times higher than that in healthy controls. On the other hand, the occurrence of paraganglioma in patients with von Recklinghausen’s disease is very rare. According to a study by Takayama et al. [12] in 2001, only five cases have been reported in Japan. Given that no additional case reports have been published since then, this is indeed an extremely rare condition. To the best of our knowledge, the reason why pheochromocytoma develops more frequently than paraganglioma in patients with von Recklinghausen’s disease has not been reported. Furthermore, there are no reported von Recklinghausen’s disease cases of the resection of liver metastases long after the initial primary paraganglioma resection. Actually, considering the long interval between resection of the retroperitoneal paraganglioma to the identification of the liver tumors, there may be a possibility that the paragangliomas in the liver were primarily arising from liver. However, as mentioned below, the long interval is not uncommon in cases with liver metastasis from retroperitoneal paraganglioma. Furthermore, to the best of our knowledge, there have been no reports regarding primary liver paraganglioma. At last, the histopathological findings of the extra-adrenal retroperitoneal paraganglioma and liver paragangliomas were similar. Taken together, the liver tumors in the case were judged as metastases from the extra-adrenal retroperitoneal lesion. We, therefore, considered the von Recklinghausen’s disease case particularly important.

Studies have reported that distant metastasis is not uncommon in paragangliomas irrespective of the presence of von Recklinghausen’s disease. Sclafani et al. [8] reported that 11 of 22 retroperitoneal paragangliomas showed distant metastases. It is also of note that the time to recurrence is longer. For example, we previously reported a case of liver metastasis 9 years after the initial surgery [13]. Lack et al. [14] also reported a case of metastatic paraganglioma 25 years after the initial treatment. In addition, Sclafani et al. [8] reported that the time to recurrence was 7 years or longer in two of the 11 patients. To the best of our knowledge, there has been no case report of metastases from paraganglioma in patients with von Recklinghausen’s disease. To date, it is unclear whether the characteristics of the tumors are the same as those of metastatic tumors from paragangliomas in patients without von Recklinghausen’s disease. Nevertheless, we speculate that the characteristics of paragangliomas in the present case may be different from the characteristics of those without von Recklinghausen’s disease, considering that pheochromocytoma occurring in patients with von Recklinghausen’s disease was more associated with malignancy than those without von Recklinghausen’s disease [13]. We are continuing to follow up this patient for possible recurrence. We also believe that the benefits of surgical treatment of metastatic lesions need to be ascertained by following up the patient over time.

## Conclusions

We report a resected case of liver metastases from extra-adrenal retroperitoneal paraganglioma in a patient with von Recklinghausen’s disease 16 years after the initial surgery.

## Consent

Written informed consent was obtained from the patient for publication of this case report and any accompanying images.
